# Design and Evaluation of a Low-Cost Smartphone Pulse Oximeter

**DOI:** 10.3390/s131216882

**Published:** 2013-12-06

**Authors:** Christian L. Petersen, Tso P. Chen, J. Mark Ansermino, Guy A. Dumont

**Affiliations:** 1 Department of Anesthesiology, Pharmacology and Therapeutics, University of British Columbia, Vancouver, BC V6T 1Z3, Canada; E-Mails: peter.chen@cw.bc.ca (T.P.C.); anserminos@yahoo.ca (J.M.A.); 2 Department of Electrical and Computer Engineering, University of British Columbia, Vancouver, BC V6T 1Z4, Canada; E-Mail: guyd@ece.ubc.ca

**Keywords:** pulse oximetry, mHealth, telemonitoring, pneumonia diagnostics

## Abstract

Infectious diseases such as pneumonia take the lives of millions of children in low- and middle-income countries every year. Many of these deaths could be prevented with the availability of robust and low-cost diagnostic tools using integrated sensor technology. Pulse oximetry in particular, offers a unique non-invasive and specific test for an increase in the severity of many infectious diseases such as pneumonia. If pulse oximetry could be delivered on widely available mobile phones, it could become a compelling solution to global health challenges. Many lives could be saved if this technology was disseminated effectively in the affected regions of the world to rescue patients from the fatal consequences of these infectious diseases. We describe the implementation of such an oximeter that interfaces a conventional clinical oximeter finger sensor with a smartphone through the headset jack audio interface, and present a simulator-based systematic verification system to be used for automated validation of the sensor interface on different smartphones and media players. An excellent agreement was found between the simulator and the audio oximeter for both oxygen saturation and heart rate over a wide range of optical transmission levels on 4th and 5th generations of the iPod Touch™ and iPhone™ devices.

## 1. Introduction

Every year, millions of young children die of common diseases such as pneumonia and diarrhea [1], in most cases due to the onset and progression of an inflammatory state of the body called sepsis. Sepsis affects the ability of the lungs to transfer oxygen to the hemoglobin molecules in the blood, which is essential for the function of cells in the body. A short interruption in the supply of oxygen will impair cellular function, and a sustained interruption will rapidly cause cellular injury and eventually death. Detection of reduced oxygen levels in the blood is therefore a key indicator of patients requiring immediate intervention.

Pulse oximetry is a non-invasive optical sensing technology that is able to measure arterial oxygen saturation. This technology has contributed significantly to reducing the risk of death associated with anesthesia and surgery. The pulse oximeter has become a standard monitoring device in modern hospitals [2,3], mandatory in North America, much of Europe and many other regions around the world. However, there are still locations globally where pulse oximeters are not routinely used during anesthesia, as they are not available, and an estimated 77,000 operating rooms worldwide are without oximeters [4]. The World Health Organization (WHO) is addressing this shortfall through the Global Oximetry (GO) initiative [5,6]. As a result, representatives of the Association of Anesthetists of Great Britain and Ireland, the WFSA, and the Harvard School of Public Health, have created a charity called the “Lifebox” to facilitate access to low-cost pulse oximeters suitable for use in anesthesia [7]. The Lifebox oximeter is supplied for US $250 and supported by international donations.

The pulse oximeter also has the potential to act as a diagnostic device in respiratory [8] and cardiac diseases [9], as well as systemic diseases such as pre-eclampsia and sepsis that affect multiple body systems including the lungs [10,11].

A pulse oximeter works by shining light from two Light Emitting Diodes (LEDs) at different wavelengths, typically 660 nm (visible red) and 910 nm (near infrared), through the arterial blood of a finger or an ear and detecting the transmitted light with a photodiode. Hemoglobin molecules with and without oxygen attached have different optical absorption characteristics at these wavelengths, and the oxygen saturation, SpO_2_, can be deduced from the ratio of the transmitted light at the two wavelengths. SpO_2_ is the percentage of hemoglobin molecules that have oxygen attached compared to those that are not bound to oxygen.

A healthy individual has an oxygen saturation level above 95%. A decrease below 95% is a strong indicator of an oxygen delivery or consumption imbalance, for example caused by impeded gas exchange in the lungs resulting from severe respiratory diseases like pneumonia and asthma [12–15] or due to an increase in consumption as well as impeded gas exchange seen in other systemic inflammatory and infectious diseases [10]. In this way, pulse oximetry can for example be used to differentiate severe pneumonia from the common cold or other mild infections.

Pulse oximetry therefore has the potential of being a powerful tool in the prevention of childhood mortality in low- and middle-income countries. Unfortunately, these areas of the world remain largely without access to the technology. Part of the problem is that conventional pulse oximeters are expensive and bulky devices intended for use in modern hospitals, and are unsuited for use in resource low settings [16–18].

In order to make pulse oximetry more available we have previously developed a so-called Phone Oximeter [19], that interfaces a commercial microcontroller-based pulse oximeter module with a smartphone. Phones are widely available even in the most remote areas [20], and have become a cornerstone in developing economies and the livelihood of people everywhere. For example, Africa has seen a tremendous growth in mobile phone usage in recent years, with 648.4 million mobile phone subscriptions in 2011, more than in the United States or the European Union [21]. Furthermore, the smartphone portion of the mobile market is set to surpass that of basic and feature phones, driven mainly by the growth in the emerging markets [22].

**Figure 1.**
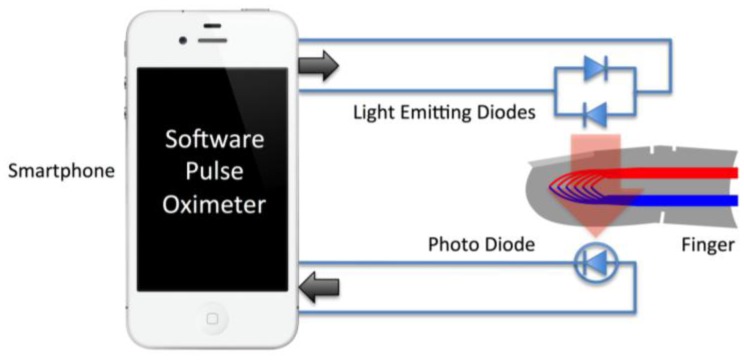
Principle of the low-cost smartphone oximeter. An oximeter finger sensor with two light emitting diodes and a photodiode is interfaced to a smartphone running a software pulse oximeter application.

Usability studies of the Phone Oximeter prototype previously undertaken both in Canada and Uganda gave overall usability scores of 82% and 78% respectively, indicative that a phone can be a functional oximeter interface [23]. The use of the phone as the display and power source of the pulse oximeter can overcome some of the challenges of distributing the technology, but the microcontroller oximeter modules are still prohibitively expensive. In this paper we describe the further development of a low-cost smartphone-based oximeter that requires no intermediate microcontroller, interfacing the sensor directly to the phone (Figure 1). By leveraging the full capabilities of the phone in this fashion, the total cost of the new device is reduced to that of the finger probe itself, and all supporting infrastructure is inherent to the host mobile phone. A clinical oximeter finger probe can be manufactured for almost two orders of magnitude less than the price of the not-for-profit Lifebox oximeter, thus potentially giving the Phone Oximeter significant global reach.

Any viable implementation of a clinical sensor that relies on consumer electronics must have an effective way of verifying performance across different devices. We present an automatic simulator-based test system that can be used to systematically examine the entire clinically relevant range of operation of the low-cost smartphone oximeter and validate the system across many different smartphone hardware versions.

## 2. Experimental Setup

### 2.1. Sensor Interface

A conventional oximeter sensor contains two LEDs for actuation and a photodiode for detection (Figure 1). The audio interface of any phone or smartphone is well suited to drive such a sensor. The audio interface has a high-current output capable of driving the low impedance load of the LEDs and a high-gain input designed to interface to a high-impedance Junction-gate Field Effect Transistor (JFET) electret microphone pre-amplifier, equally suitable for amplifying the photodiode signal.

**Figure 2.**
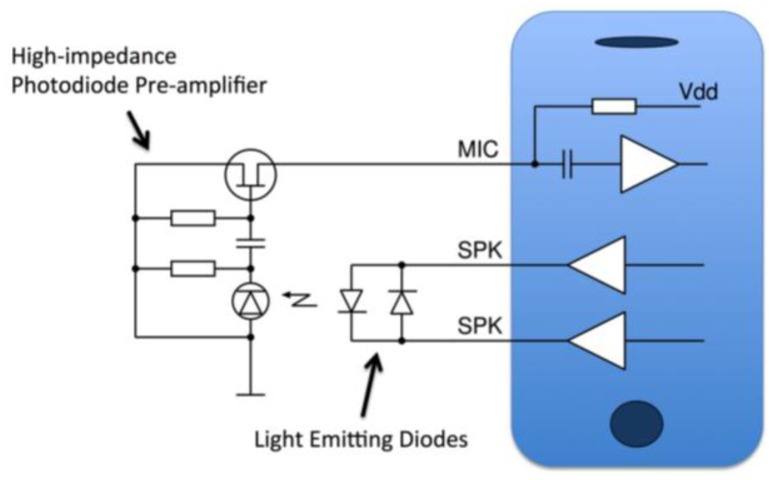
Schematic interface of a low-cost smartphone oximeter. The LEDs are driven by the headset speaker output and the photodiode signal is amplified by a line-powered JFET amplifier before being detected by the microphone.

The sensor LEDs of the audio-based smartphone oximeter are driven directly by the speaker output of the phone (Figure 2). The LEDs are wired in reverse polarity to facilitate alternating activation at opposite polarities of a driving signal. With the peak-to-peak amplitude of the speaker output larger than the forward voltage threshold of the LEDs, this can be accomplished by sending a suitable audio signal to the speaker output. The forward voltage thresholds of the red and infrared diodes are approximately 1.3 and 1.8V, respectively. The Apple iOS family of mobile devices (iPhone, iPod Touch, iPad and iPad Mini) was found to generate sufficient output voltages to perform clinical measurements.

The oximeter sensor photodiode was interfaced to the microphone pin of the phone with an un-biased AC-coupled JFET photodiode preamplifier circuit (Figure 2). A straight connection of the photodiode directly to the pin was also found to produce a limited clinical measurement, but the low-transmission resolution was unsatisfactory. The unbiased photodiode pre-amplifier configuration was chosen because it has the optimal signal to noise ratio, and sufficient bandwidth to handle the signals from the LEDs. The pre-amplifier is powered by the line power present on the microphone pin. The phone microphone pin is connected through an internal resistor to the power supply of the phone, to facilitate driving a JFET in conventional electret microphones. The oximeter input channel thus closely resembles that of the conventional electret microphone.

### 2.2. Software Interface

Since no external oximeter microcontroller module is used, the low-cost smartphone oximeter must perform the signal processing necessary to calculate the oxygen saturation from the raw photodiode signal and to interface inputs and outputs to the user. This has been implemented within a single pulse oximeter software application running on the smartphone. The pulse oximeter application has been realized in-house using our internally developed cross-platform development environment.

**Figure 3.**
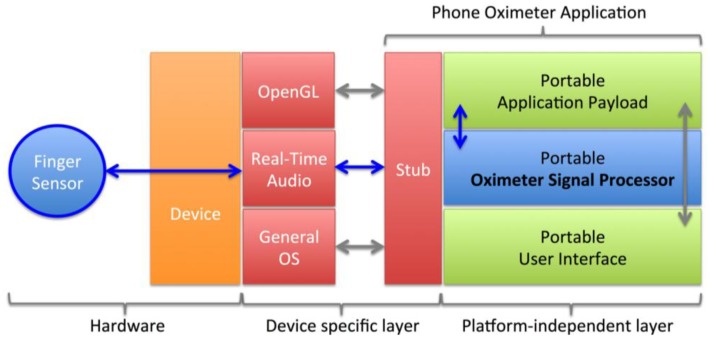
Structure of the audio-based smartphone oximeter application.

The application consists of a portable payload linked to a system-dependent stub that launches the application and is responsible for relaying system events to the payload (Figure 3). Data is transferred between the sensor and the application through the real-time audio layer of the smartphone to a portable signal-processing unit and OpenGL based user interface. The real-time audio layer was based on the AudioUnit framework on iOS-based devices and OpenSL on Android-based devices. These interfaces are commonly used for Voice Over IP (VOIP) applications requiring full-duplex real-time communication. The oximeter signal processing routines were written in portable C for maximum performance and portability between devices and architectures.

**Figure 4.**
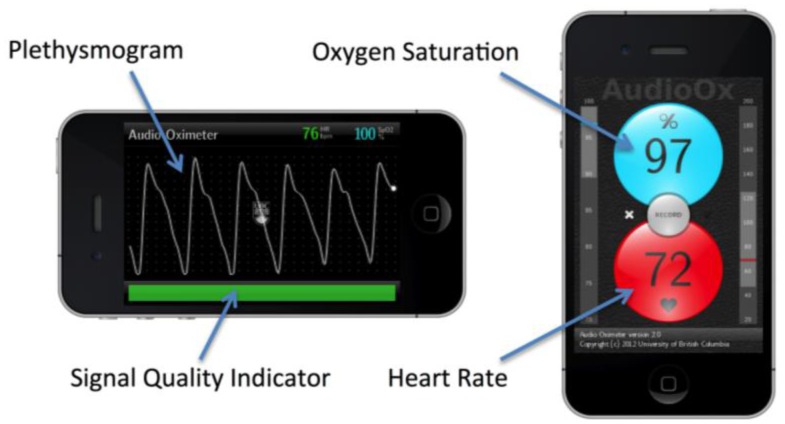
Interface of the smartphone oximeter software application. The landscape orientation features a photoplethysmogram and signal quality indicators, and the portrait orientation shows the detected values of oxygen saturation and heart rate.

The audio oximeter graphical user interface was designed to show key elements required for pulse oximetry interfaces (Figure 4). The user interface automatically reconfigures according to the orientation of the device. In landscape (horizontal) mode, a photoplethysmogram is displayed in real time, updating with the refresh rate of the display, typically 30–60 Hz. The top left shows the current detected values of oxygen saturation and heart rate, and the bottom bar displays indicators of the signal quality and the state of the signal-processing algorithm.

In portrait (vertical) mode, the currently detected oxygen saturation and heart rate values are prominently displayed. The oxygen saturation background color changes gradually from light blue at a normal saturation to purple at low saturation to augment the numerical feedback. Similarly the heart rate indicator shades its red background color in synchronization with the heartbeat to provide an intuitive feedback to the user about the heart rate range. Vertical scales to the left and right of the trend indicators mark the current trend values in a range with preselected thresholds, allowing the operator to relate the current values to a population average. A record button in the center of the display facilitates recording data to the solid-state storage of the device for subsequent data analysis.

### 2.3. Oximeter Signal Processing

**Figure 5.**
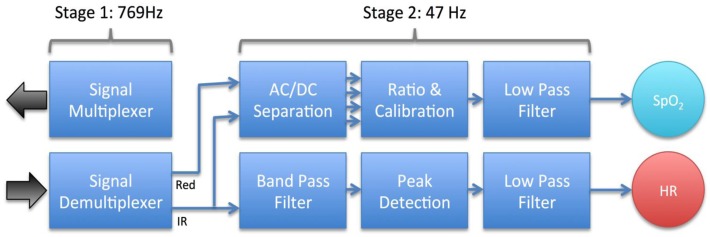
Diagram of the signal processing chain of the audio-based smartphone oximeter.

The signal processing chain of the audio-based smartphone oximeter includes a signal multiplexer for generating the LED signals, a demultiplexer for recovering the interlaced input signal, and algorithms and filtering for extracting oxygen saturation and heart rate (Figure 5). Unique to the smartphone implementation is a sampling rate of 8,000 Hz, substantially faster than any conventional oximeter implementation, and an AC-coupled sensor interface. Conventional oximeter sensor interfaces are DC-coupled and rely on sampling sustained input signal levels.

The first stage of the oximeter sends an audio output signal to the LEDs with alternating polarity. The resulting pulse train of red and infrared light passes through the small blood vessels of a finger and is picked up by the photodiode. The photodiode signal contains a series of interlaced peaks due to the two different wavelength of light, which is de-multiplexed to isolate a signal from each diode, and down-sampled to increase signal quality and reduce processing overhead. The resulting raw signals from the red and the infrared diodes are then passed to the second stage of the signal processing chain, responsible for extracting the oxygen saturation and heart rate from the raw diode signals.

In the second stage of signal processing, the oximeter ratio *R* is determined from:

(1) $$R=\frac{AC_{\text{Red}}/DC_{\text{Red}}}{AC_{\text{IR}}/DC_{\text{IR}}}$$

where *AC* and *DC* refers to the AC and DC components of the red and infrared (IR) diode signals. *DC* is determined as a average over a window of approximately eight heartbeats, and *AC* as the Root-Mean-Square (RMS) amplitude of the signal calculated over the same interval:

(2) $$\begin{array}{c} DC_{\text{Red}}=\frac{1}{N}\overset{N}{\underset{i=0}{\text{∑}}}{\text{Red}}_{i}\\ AC_{\text{Red}}=\sqrt{\frac{1}{N}\overset{N}{\underset{i=0}{\text{∑}}}{\left({\text{Red}}_{i}-DC_{\text{Red}}\right)}^{2}} \end{array}$$

where *N* is the number of samples in the interval, *i* is the sample index, and *Red_i_* a sample of the demultiplexed signal originating from the red LED. Using moving averages to determine the DC and AC signal amplitudes is a simple and robust solution with very little overhead. The AC values in Equation (1) are conventionally determined from a difference between signal maximum and minimum. The alternative quadratic mean expression in Equation (2) provides a statistical measure of AC that was found to have comparable noise rejection and less complexity.

Once the ratio *R* has been calculated, the corresponding oxygen saturation is determined. The oxygen saturation is approximately linearly related to *R*, but an accurate conversion requires an empirical relationship between *R* and oxygen saturation to be determined through invasive blood gas measurements and stored in a calibration lookup table [24]. The oxygen saturation is finally fed through a low-pass filter to provide a stable reading for the application display.

The heart rate was determined by feeding the IR diode signal, which is generally the strongest of the two wavelengths, through a band pass filter, performing simple polarity based peak detection, and calculating the time between peaks of the same polarity. Simple branching logic is used to drop noisy signals where the rate is unphysical. The raw heart rate is passed through a low pass filter to generate a stable reading for the application display.

### 2.4. Test and Verification

In a prior first test of an audio-based oximeter, synchronous readings from a conventional calibrated oximeter were compared to the audio oximeter in a hypoxic environment. With ethics board approval and written consent this was accomplished by recording paired oximeter readings from nine subjects in a hypoxia chamber by interfacing a commercial microcontroller-based oximeter to an iPhone also running the audio-based oximeter [25]. The volunteers wore both sensors on the non-dominant hand and had unrestricted movement during the hypoxic exposure. Data with artifacts in either sensor reading were discarded, resulting in more than 21 h of valid per-second paired readings. The results showed good agreement between the two oximeters, and the RMS accuracy was within the ISO standard range of 4% [26] in the study population over the clinical range of oxygen saturation (70%–100%).

However, carrying out human studies under low oxygen conditions carries a risk of complications, and cannot ethically involve high-risk individuals such as children and patients with severe cardiovascular diseases, that would represent cases with limited perfusion and transmission. Human studies are also time- and resource intensive, and it would be impractical to test on humans with every hardware generation of smartphones. As a case in point, there are more than 25 different hardware generations in the Apple family of mobile devices at the time of writing. Testing the audio-based oximeter against all of these devices in human trials is not feasible. Once the sensor has been validated according to the standard requirements on a reference device, new means for testing are needed to ensure equivalent performance on other devices.

**Figure 6.**
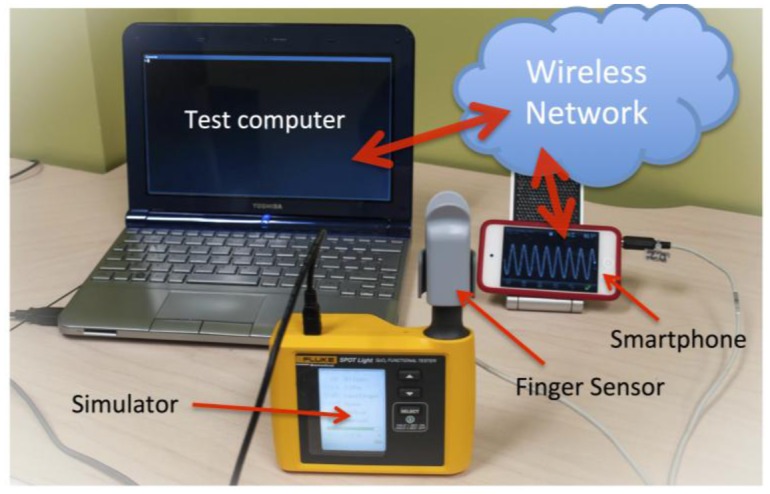
Oximeter simulator test setup for evaluating the audio oximeter device.

For these reasons we have developed a simulator-based test system (Figure 6). The advantage of using a patient simulator is that the full range of skin pigmentation and perfusion levels can easily be evaluated [27], and many different devices can be systematically tested using a degree of automation. The setup consists of a Fluke SpotLight ^™^ oximeter simulator, controlled by a computer through the serial interface of the simulator. The audio-based oximeter application described above was extended to provide a web service on port 8080 of the WiFi-connected phone, and to continuously post the current oxygen saturation and heart rate readings on this server. Scripts running on the computer pull the readings from the server over the network while the simulator settings are being controlled. The performance of the oxygen saturation and heart rate algorithms can now be evaluated automatically by setting the oximeter ratio and heart rate on the simulator, waiting for the oximeter algorithms to settle, recording the readings from the server, and repeating the measurements over the entire clinical range.

## 3. Results and Discussion

**Figure 7.**
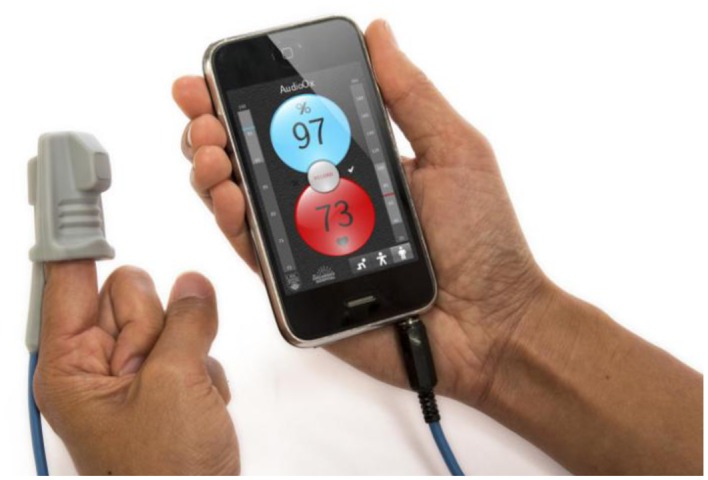
Low-cost audio-based smartphone oximeter with integrated pre-amplifier running on a 3rd generation iPhone device with an ACare commercial clinical finger sensor.

All prototyping, testing and trials of the audio oximeter were performed with Apple iPhone and iPod Touch devices running the iOS operating system. The oximeter pre-amplifier (Figure 2) was integrated into the 3.5 mm Tip-Ring-Ring-Sleeve (TRRS) connector used to connect the oximeter finger sensor to the phone audio jack (Figure 7). Multiple finger sensors from Nellcor, Nonin and Envisen were evaluated and found to perform adequately under simulated conditions. The Nellcor and Envisen sensors had the best signal strength due to larger area of the photodiode.

**Figure 8.**
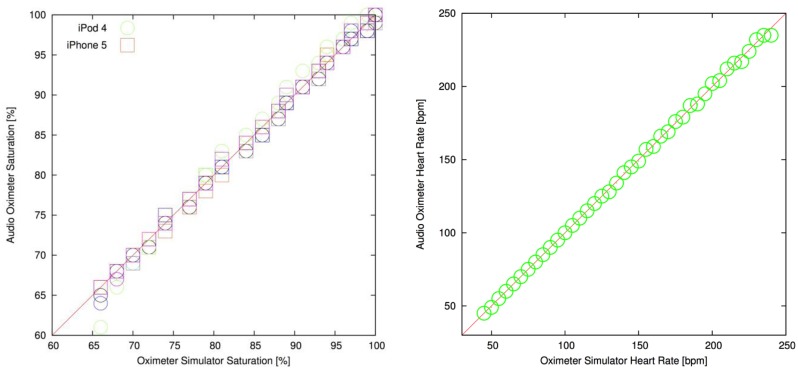
Correlation between the audio oximeter and patient simulator for oxygen saturation and heart rate. Oxygen saturation for six different transmission levels for both the iPod Touch 4 (circles) and iPhone 5 (squares) is shown.

The oximeter application was evaluated with systematic tests for oxygen saturation and heart rate against the patient simulator (Figure 8, Table 1). The results show a strong correlation between heart rate and oxygen saturation values selected on the simulator. For oxygen saturation, the tests were repeated at optical transmission levels of 5, 10, 50, 100, 200 and 300 parts per million (ppm), which represents a large range of skin pigmentations. The same per-generation calibration lookup was used at all transmission levels on the two devices. The overall Pearson correlation coefficients for the oxygen saturation measurements were 0.9976 and 0.9992 for the iPod Touch 4 and the iPhone 5, both indicative of excellent correlation (a correlation of 1.0 representing an ideal straight line). The RMS accuracies of the oxygen saturation readings over all transmissions were 0.85% for the iPod Touch 4 and 0.45% for the iPhone 5 with a bias of 0.12% and 0.05%, respectively. Transmission levels or hardware revisions did not affect the heart rate algorithm, and the simulator was well correlated with the device (Pearson correlation coefficient was 0.9997).

## 4. Conclusions and Outlook

We have developed a new low-cost audio-based smartphone oximeter that interfaces a standard clinical finger sensor to the audio port of a smartphone through a line-powered high-impedance preamplifier integrated into the sensor connector. The oximeter relies on the internal analog audio interface of the phone to drive the sensor, and all signal processing is implemented in a standard software application that can be downloaded to the phone using the conventional app stores. This solution eliminates almost all of the hardware costs of a conventional oximeter, and allows a smartphone to be used as a diagnostic tool for diseases such as pneumonia that could save the lives of millions of children in low and middle-income countries.

The number of smartphones on the market today is staggering, and represents an ever-moving target for developers. For example, the Google app store currently supports more than 3,500 Android based smartphones, and new devices are arriving constantly. It is unfeasible to test any clinical sensor on all of these devices through human trials, and solving this challenge is essential to the success of sensor-based mHealth applications in general. With the advances of mobile technology and personal health, new methods and technologies are needed to effectively test medical mobile technology without human trials. The solution lies in validating the sensors in human studies using a reference device and then developing automated patient simulator systems that are verified and proven to be physiologically representative.

We have developed an automated test system based on a commercially available simulator, and found that the oxygen saturation and heart rate output of our device has excellent correlation and low RMS error compared with the simulator on both iPod Touch and iPhone devices. More work is needed to test other hardware generations and investigate variability within each generation. We continue this work with the goal of developing a robust automatic oximeter simulation system that can be used to systematically investigate device-to-device variability and allow smartphone-based low-cost oximeters to be widely deployed across different mobile platforms and device hardware generations.

## Figures and Tables

**Table 1.** Accuracy and correlation of tests at different simulator transmission settings.

	**iPhone 5**			**iPod Touch 4**		

Transmission	A_RMS_ [%]	Bias [%]	Correlation	A_RMS_ [%]	Bias [%]	Correlation
300 ppm	0.38	−0.14	0.9995	0.65	0.43	0.9990
200 ppm	0.38	0.05	0.9995	0.31	−0.10	0.9997
100 ppm	0.38	−0.05	0.9994	0.44	0.19	0.9995
50 ppm	0.22	0.05	0.9998	0.65	0.43	0.9992
10 ppm	0.62	0.19	0.9986	0.69	0.19	0.9993
5 ppm	0.62	0.19	0.9992	1.65	−0.43	0.9944
